# M. Dumas on the Chemistry of Organized Beings

**Published:** 1842-11-19

**Authors:** 


### ANALECTA.

*M. Dumas on the Chemistry of Organized Beings.*—It appears that MM. Dumas and Boussingault, in France, have been following out nearly the same pursuits in Organic Chemistry, as Professor Liebig has been doing in

Germany. It is immaterial to us to whom the priority belongs; the important point for science is, that these gentlemen have arrived at very similar conclusions.

From a recently published memoir of M. Dumas, we shall select a few passages that bear more immediately on the physiology of plants and animals.

"We have," says he, "considered *plants* as constituting an immense reducing or decomposing apparatus, that is nourished by carbon, hydrogen, and nitrogen—derived from the decomposition of carbonic acid, water, and oxide of ammonium—and *animals* as forming a large consuming apparatus, in which there is constantly going on in the combustion of these elements, carbon, hydrogen, and ammonium, to form these very compounds.

We have laid it down as a recognized principle that plants form, or prepare, from mineral substances the organic materials of their composition, that these materials are taken into the bodies of animals, are there subjected to the process of digestion, thus become animalized, and are subsequently again brought back by a vital process to the state of mineral and inorganized matter.

As accessory results of our researches, we may state that some plants absorb a certain portion of nitrogen from the atmosphere, while others do not; that animal heat is owing solely to respiration; that the chemical process of this function (respiration) takes place not in the lungs but in the capillary vessels of the whole body; that digestion effects two important results, viz. the assimilation of azotised matter, and the restitution of combustible matters to the blood."

M. Dumas then discusses at considerable length, the striking effects of ammonia in promoting vegetation; this seems to be the potent agent in most manures. Hence whatever tends to assist the formation of this substance, or to render it more fixed and abiding, is found to increase the valuable properties of manures. The addition of a solution of sulphate of iron, or of weak sulphuric acid has this effect, and has been found to add greatly to the fertilizing properties of these matters. A sulphate of ammonia is formed, and remains fixed; the salt being not volatile like its alkaline bases.

With respect to the much vexed question, as to the source of animal heat, it seems to be the opinion of M. Dumas, that during each act of respiration a certain portion of oxygen is absorbed directly into the blood, and a certain portion of carbonic acid—already formed and existing in the blood—displaced and evolved. There is no direct union or combustion, so to speak, of hydrogen or carbon with oxygen in the lungs, as imagined by Lavoisier and Laplace; the formation of the carbonic acid seems to be a slow and successive act that is constantly going on in the minute blood-vessels; and the venous blood, when it reaches the right side of the heart, is already charged with it, and ready to give it off when exposed to the air in the cells of the lungs.

It is necessary to keep in mind that the production of animal heat, the exhalation of carbonic acid into, and the disappearance of oxygen from, the respired air are three separate phenomena—connected, indeed, the one with the other, but not implying that they are of simultaneous occurrence. Or we may express our meaning in different words thus: the generation of animal heat, the decarbonization, and the subsequent arterialization, of the blood, are three mutually-associated, but not coincident, phenomena. The blood becomes arterialized without any necessary production of heat at the time: and the gradual formation of carbonic acid—an act which is necessarily attended with the evolution of caloric—is going on in every capillary vessel throughout the body.

"Respiration," says our author, "introduces oxygen into the blood and renders it of a bright red colour; carbonic acid is at the same time expelled from it.

*L'oxygene absorbé sert à bruler du lactate de soude, et en general des sels de soude. L'acide lactique transforme celui-ci en lactate et degage acide carbonique. Cet acide lactique provient des alimens sucrés ou amylacés.*"

"That fatty matters also form salts with the soda of the blood. When they are in excess, they become deposited around the vessels through which they have exsuded, and, by being blended with the albuminous fluids in a state of repose, they serve for the production of adipose cellules or vesicles, and the animal becomes fat—in other words, it lays up a supply of combustible matter. When the organized materials of the blood are consumed (brulés) without being replaced, the vital fluid becomes more decidedly alkaline and begins to react upon the adipose vesicles which surround the vessels; the fat and albumen of the cellules is re-dissolved and absorbed by the blood, and the animal becomes emaciated—in other words, it consumes the combustible matter which had been stored up at a former time."

M. Dumas closes his remarks on this subject by pointing out the difference in the various kinds of food, according to their mode of action on the system, and the changes they undergo during digestion and assimilation. He arranges them in three classes:—

1. Aliments of assimilation—viz. fibrine, albumen, and caseum: these are all of a highly azotised nature.

2. Soluble non-azotised aliments of respiration; such as starch, sugar, acid or acidifiable substances, &c., which at once undergo combustion in presence of the soda in the blood;—hence the production of heat manifested from the very commencement of their digestion.

3. Aliments of respiration, insoluble and therefore capable of being stored up in the body, viz.: various kinds of fatty matter."—*Annales de Chimie.*

We may here insert a paragraph or two from M. Liebig's lecture on the different kinds of food.

"Another most interesting result of M. Liebig's researches is that vegetable albumen, fibrine, and caseine, not only have the same properties as the corresponding elements derived from animal matters, but also exhibit their azote and carbon in the same relations to each other. Thus chemical analysis shews us that herbivorous animals find the constituent materials of their blood, their albumen and their fibrine, already prepared in plants; and that the juice of plants, the vegetable albumen, the farina of wheat and of other *cerealia* contain the principle of muscular fibre, while lentiles, peas and beans, contain the same azotised substance that is present in milk. They (herbivorous animals) live upon the flesh, blood, and cheese supplied them by plants; while, on the other hand, their flesh and blood serve for food to the carnivorous tribe. There is thus a complete identity between the azotised principles existing in vegetables and those in animal substances. Their chemical properties are alike; for we find vegetable albumen, obtained by boiling the juice of plants, and freed from all fatty and colouring matter by means of ether and alcohol, can scarcely be distinguished from the white of eggs."—*Medico-Chirurgical Review*. Oct. 1842. *From Algemeine Zeitung*.

